# Spns2 Transporter Contributes to the Accumulation of S1P in Cystic Fibrosis Human Bronchial Epithelial Cells

**DOI:** 10.3390/biomedicines9091121

**Published:** 2021-08-31

**Authors:** Aida Zulueta, Michele Dei Cas, Francesco Luciano, Alessandra Mingione, Francesca Pivari, Ilaria Righi, Letizia Morlacchi, Lorenzo Rosso, Paola Signorelli, Riccardo Ghidoni, Rita Paroni, Anna Caretti

**Affiliations:** 1 Biochemistry and Molecular Biology Laboratory, Department of Health Sciences, University of Milan, 20142 Milan, Italy; aida.zulueta@guest.unimi.it (A.Z.); francesco.luciano2@studenti.unimi.it (F.L.); alessandra.mingione@unimi.it (A.M.); francesca.pivari@unimi.it (F.P.); paola.signorelli@unimi.it (P.S.); riccardo.ghidoni@unimi.it (R.G.); 2 Clinical Biochemistry and Mass Spectrometry Laboratory, Department of Health Sciences, University of Milan, 20142 Milan, Italy; michele.deicas@unimi.it (M.D.C.); rita.paroni@unimi.it (R.P.); 3 Thoracic Surgery and Lung Transplant Unit, Fondazione IRCCS Ca’ Granda Ospedale Maggiore Policlinico, 20122 Milan, Italy; ilaria.righi78@gmail.com (I.R.); lorenzo.rosso@unimi.it (L.R.); 4 Respiratory Unit and Cystic Fibrosis Center, Internal Medicine Department, Fondazione IRCCS Ca’ Granda Ospedale Maggiore Policlinico, 20122 Milan, Italy; letizia.morlacchi@gmail.com; 5 Department of Pathophysiology and Transplantation, University of Milan, 20122 Milan, Italy

**Keywords:** Spns2, S1P, sphingosine, SphK1, SGPL1, cystic fibrosis, sphingolipid, lung

## Abstract

The role of S1P in Cystic Fibrosis (CF) has been investigated since 2001, when it was first described that the CFTR channel regulates the inward transport of S1P. From then on, various studies have associated F508del CFTR, the most frequent mutation in CF patients, with altered S1P expression in tissue and plasma. We found that human bronchial epithelial immortalized and primary cells from CF patients express more S1P than the control cells, as evidenced by mass spectrometry analysis. S1P accumulation relies on two- to four-fold transcriptional up-regulation of SphK1 and simultaneous halving of SGPL1 in CF vs. control cells. The reduction of SGPL1 transcription protects S1P from irreversible degradation, but the excessive accumulation is partially prevented by the action of the two phosphatases that are up-regulated compared to control cells. For the first time in CF, we describe that Spns2, a non-ATP dependent transporter that normally extrudes S1P out of the cells, shows deficient transcriptional and protein expression, thus impairing S1P accrual dissipation. The in vitro data on CF human bronchial epithelia correlates with the impaired expression of Spns2 observed in CF human lung biopsies compared to healthy control.

## 1. Introduction

Cystic fibrosis (CF) is a recessive congenital disease that affects 75,000 patients worldwide, highly prevalent among Caucasians. It is caused by the mutation of the chloride/carbonate channel CFTR (cystic fibrosis transmembrane conductance regulator) [1] and induces dysfunction of multiple organs, primarily the lungs and pancreas. CF patients suffer from chronic inflammation and are unable to clear airways infections [2]. A growing body of evidence indicates that sphingolipids (SPL) highly contribute to regulating pulmonary infections, inflammation, immunity responses in CF [3,4,5,6] and the development of fibrosis [7]. Among the various SPL, the role of S1P (sphingosine 1 phosphate) in CF has been investigated since 2001, when Boujaoude L.C. and colleagues described that the CFTR channel regulated the inward transport of S1P [8]. From that on, various studies associated F508del CFTR, which represents the most frequent mutation in CF patients, with altered expression of tissue [9] and plasma [10] S1P. S1P supplementation restores the functional expression profile in CF defective dendritic cells [11] and inhibition of S1P degradation reduces CF enhanced bronchial endothelial monolayer permeability [12] and ameliorates lung defense from infection [9]. S1P derives from the phosphorylation of sphingosine (Sph), the backbone of SPL, utilizing two kinases: SphK1 and SphK2. These two mammalian isoforms display different catalytic properties and subcellular localization: SphK1 is mainly cytosolic, while SphK2 is mostly found in the plasma membrane, endoplasmic reticulum (ER), mitochondria, and nucleus [13]. Two degradation processes finely regulate the amount of intracellular S1P: dephosphorylation by S1P phosphatases and cleavage by S1P lyases. The S1P phosphatases (SGPPs) remove the phosphate group driving S1P back to Sph while the S1P lyase (SGPL) cleaves S1P in its precursors [13]. Once synthesized, S1P acts on intracellular targets and is involved in NF-kB (nuclear factor-kappa B) activation [14,15]. However, it is also extruded in the extracellular compartment, where it activates autocrine and paracrine signaling by binding to G-protein coupled S1P receptors on cell surfaces (S1P1–5) [16]. In vitro and in vivo studies have demonstrated that besides the ABC transporters, the Spinster homolog 2 (Spns2) is responsible for transporting S1P [17,18]. Spns2 is a member of the major facilitator superfamily of non-ATP dependent transporters. Two independent research groups first discovered in zebrafish a correlation between a mutation in Spns2 and a defect in myocardial precursors’ migration, thus leading to heart malformation [19,20]. In vitro studies and Spns2-KO mice models show that Spns2 transport function is crucial for maintaining lymph and serum S1P levels and consequently allowing adequate circulation of lymphocytes [21,22]. As recently reviewed by Spiegel and colleagues [23], Spns2-mediated S1P transport is involved in immune cell trafficking and functions, hence emphasizing its role in inflammatory, adaptive immune-related pathologies and metastatic cancer. S1P is a bioactive signaling molecule that influences cell migration, growth and differentiation besides tissue repair. It has been reported that S1P metabolism plays a role in inflammatory lung disease and fibrosis. S1P and its analogue FTY720 (Fingolimod) significantly decrease pulmonary inflammation in mouse and rat models of acute lung injury [24,25]. Previous studies in CF humans and CF mutant mice have shown that Sph/S1P pathway is dysregulated thus contributing to CF pathology through modulation of inflammation, airway plugging, and tissue remodeling [26]. Xu Y and colleagues reported that low S1P in the CF lung milieu impairs the functionality of lung dendritic cells, thus increasing the susceptibility to pulmonary infections [11]. In the in vitro model, CFTR was demonstrated to uptake S1P from the extracellular compartment, reducing S1P activity [8]. In turn, S1P signaling is suggested to transiently and rapidly depress CFTR channel activation, hence amplifying S1P-stimulated signals [27]. In the present manuscript, we used human bronchial epithelial immortalized and primary cell lines to investigate the modulation of the Spns2 transporter together with the main enzymes driving S1P metabolism in CF versus healthy control cells. We found that S1P accumulation in CF cells is supported by an altered equilibrium between synthesizing and degrading enzymes together with a relevant impairment of Spns2 expression.

## 2. Materials and Methods

### 2.1. Reagents and Antibodies

The following materials were purchased: LHC Basal, LHC-8 w/o gentamicin culture media from Gibco (CA, USA; cat n°12679-015); FBS (cat n°ECS0180L), MEM (cat n°ECB2071L) and DMEM (cat n°ECM0728L) from Euro Clone Life Science Division (Milan, Italy); protease inhibitors from Roche Italia (Monza, MB, Italy); penicillin/streptomycin (cat n°ECB3001D) and Trypsin (cat n°ECB3051D) from Sigma-Aldrich (Burlington, MA, USA; Quick Start™ Bradford Dye Reagent (cat n°ECL170-S061) and Clarity™ Western ECL Blotting Substrates from BioRad (Milan, Italy); SYBR Green system from Takara (Kusatsu, Japan; cat n°RR420L); ReliaPrep™ Miniprep RNA extraction System (cat n°Z6011) and GoScript Reverse Transcription Mix (cat n°A2791) from Promega (Madison, WI, USA); synthetic oligonucleotides from Eurofins Genomics (Ebersberg, Germany). Methanol, acetonitrile, ammonium formate, acetic acid, potassium hydroxide and formic acid (all analytical grade) were supplied from Merck (Darmstadt, Germany). Water was MilliQ grade. Sphingosine 1-phosphate standard (cat n°8604928) was purchased by Avanti Polar Lipids (Alabaster, AL, USA). Primary antibodies: anti-β-actin (Sigma, Burlington, MA, USA; cat n°A5316, clone AC-74), anti-Spns2 (Gene Tex, Irvine, CA, USA; cat n°GTX45115). The secondary antibodies were from Jackson Laboratories (Bar Harbor, ME, USA). All reagents were of the maximal available purity degree.

### 2.2. Cell Lines

IB3-1 cells, an adeno-associated virus-transformed human bronchial-epithelial cell line derived from a CF patient (ΔF508/W1282X), were provided by LGC Promochem (Teddington, Middlesex, UK) and cultured in LHC-8 medium supplemented with 5% FBS, 1% penicillin/streptomycin at 37 °C and 5%. CO_2_. 16HBE14o- cells (hereafter termed HBE), a human bronchial-epithelial healthy cell line and CFBE41o cells, an immortalized cell line from a CF-patient homozygous for the ΔF508 allele, were developed initially by Gruenert [28] and provided by Galietta (TIGEM, Napoli). Both cell lines were grown in minimum essential medium (MEM) Earle’s salt, supplemented with 10% FBS and 1% penicillin/streptomycin at 37 °C and 5% CO_2_. All the cells used in this study were from passage 5–10; 8 × 10^5^ cells in 10 mL medium were plated in 100 mm tissue culture dishes to reach about 80% confluence. IB3-1 cells reached the due confluence in about 24–48 h, whereas 16HBE14o- and CFBE41o were maintained in culture for up to 72–96 h. Cells were detached by trypsin treatment for 3 min at 37 °C, then harvested and counted by Trypan Blue assay to assess that almost 85% of the cells were viable to perform the different assays.

### 2.3. Primary Human Bronchial Epithelial Cells

Primary human bronchial epithelial cells, either CF (hereafter termed CF-BE) or healthy (hereafter termed BE) were provided by the Culture Service of the Italian Cystic Fibrosis Foundation (FFC). All the CF-BE primary cells are homozygous for the ΔF508 mutation. Cells were cultured as previously reported [29]. Briefly, 2.5 × 10^5^ cells were plated in one T75 flask coated with 100 µL/cm^2^ of rat tail collagen solution and grown at 37 °C (5% CO_2_) in proliferative medium LHC9/RPMI1640 (1:1) without serum. The medium was changed every two days. All the cells used in this study were from passages 2–5. Cells were detached by trypsin treatment for 3 min at 37 °C and harvested at about 70% confluence. Both CF-BE and BE reached the due confluence in about five days. Before performing the different assays, cells were counted by Trypan Blue assay to assess that almost 85% of the cells were viable.

### 2.4. Sphingoid Long-Chain Bases Determination by LC-MS

Bronchial-epithelial primary and immortalized cell lines were scraped and washed in ice-cold PBS1X and then pelleted for ten minutes at 1000× *g* (4 °C). The pellet was resuspended in 100 μL of PBS1X with protease inhibitors and stored at −20 °C. Sphingolipid extraction was performed as already described in [30], coupling the Bligh–Dyer method with alkaline methanolysis. The clear supernatant was injected on a Shimadzu UPLC coupled with a Triple TOF 6600 Sciex (Concord, Ontario, CA, USA) equipped with Turbo Spray IonDrive. Sphingosines analysis was completed on Acquity BEH C18 column 1.7 μm, 2.1 × 100 mm (Waters, Franklin, MA, USA) by using, as mobile phase (A) 0.2% formic acid 2 mM ammonium formate water-solution and as mobile phase (B) methanol 0.2% formic acid 1 mM ammonium formate. The flow rate was 0.3 mL/min and the column temperature was 30 °C. The elution gradient (%B) was set as follow: 0–10 min (80–99%), 10–15 min (99%), 15–15.2 min (99–80%), 15.2–20 min (80%). Sphingoid long-chain bases were determined by monitoring the high-resolution transitions *m*/*z* 380.25 > 264.26 (S1P), 300.28 > 282.27 (Sph), by applying a DP of 50 eV and CE 30 ± 15 eV. Quantitative analysis was corrected for internal standard responses (sphinganine d17:0; *m*/*z* 288.28 > 270.27) and the results were expressed as pmoles/mg protein.

### 2.5. RNA Extraction and Quantitative RT-PCR

Real-time PCR was performed on RNA from bronchial-epithelial primary and immortalized cell lines. RNA extraction and reverse transcription followed the protocol previously described [31]. Human gene primer sequences for SphK1, SphK2, SGPL1, SGPP1, and GAPDH have already been published [31,32]. The primer sequences for Spns2 and SGPP2 were designed: hSpns2 Fw AACGTGCTCAACTACCTGGACA and hSpns2 Rev CCTCGGTCCTTGACCCCAAAG; hSGPP2 Fw TCCACCTTGGTGTGTCTCAG and hSGPP2 Rev AGGGTAGGTGAGGACGATGA. RT-PCRs were performed on a StepOnePlus Real-Time PCR Systems (Thermofisher; Monza, Italy). The target genes mRNA were normalized onto the GAPDH mRNA [33]. Determinations were done in triplicate.

### 2.6. Protein Extraction and Western Blotting

Total cell proteins were extracted from immortalized cell lines in RIPA buffer. Protein concentration in the lysates was measured by Quick Start™ Bradford Dye Reagent (595 nm OD). 10 μg of protein’s extracts were separated by electrophoresis and immunoblotted onto a nitrocellulose membrane as previously described [34]. Anti-Spns2 (1:500 in TBS-T) and anti-β-actin (1:5000 in TBS-T) antibodies were used. β-Actin expression was quantified for data normalization. The protein bands were revealed by chemo-luminescent horseradish peroxidase substrate by Alliance UVITEC (Cambridge, UK) and the intensity quantified by a software provided by the same system.

### 2.7. Human Lung Biopsies

Control human lung biopsies were obtained from patients who underwent lung surgery for cancer and target tissue was located in a peripheral area distant from the neoplasm. Inclusion criteria: pulmonary cancer patients over 18 years old. CF human lung biopsies were obtained from patients who underwent lung transplantation at Thoracic Surgery and Lung Transplantation Unit, Fondazione IRCCS, Ca’ Granda Ospedale Maggiore Policlinico. The target tissue was a macroscopically pathological area of lung parenchyma. The three CF patients enrolled were heterozygous for ΔF508 (F508del/G542X; F508del/R1066H; F508del/711 + 5G > A). Inclusion criteria: CF patients over 18 years old, with pathological SCL (chloride > 60 mEq/L) and on the list for a lung transplant. Exclusion criteria: patients subjected to previous organ transplant and pregnant women.

### 2.8. Immunohistochemistry and Immunofluorescence Analysis of Lung Biopsies

Immunohistochemistry and immunofluorescence were carried out on paraffin-embedded lung tissue fixed in buffered formalin. CF lung parenchyma was histologically compared to lung specimens from surgical samples of lung cancer patients taken away from the lesion. The Spns2 antibody diluted at 1:100 was used, with EDTA buffer as antigen retrieval. The reaction was detected with polymer HRP and DAB for immunohistochemistry and TRITC (Alexa, Fluor546; Thermofisher; Monza, MB, Italy) for immunofluorescence.

### 2.9. Immunohistochemistry of Human Bronchial Epithelial Immortalized Cell Lines

One hundred microliters of cell suspension were pipetted into the cytofunnel and centrifuged at 900 rpm for 5 min. The cells were fixed for 5 min in buffered formalin solution. Immunohistochemistry was performed as described for lung biopsies.

### 2.10. Statistical Analysis

For two-group comparisons, data significance was evaluated through an unpaired Student’s *t*-test with an alpha level of 0.05. When comparing more groups, a one-way ANOVA was performed with an alpha level of 0.05; if significant, it was followed by Dunnett’s multiple comparisons tests. The results from bronchial-epithelial immortalized cell lines are expressed as mean ± SEM as those from bronchial-epithelial primary cell lines. The descriptive statistics are extensively reported in the Supplementary Materials (Table S1). Western blotting and immunofluorescence images are the most representative of three individual experiments. Statistical analysis and graph illustrations have been performed by GraphPad Prism software, version number 7.00 (La Jolla, CA, USA).

## 3. Results

### 3.1. Expression of S1P and Sph in Human Bronchial Epithelial Immortalized Cell Lines

**Figure 1:**
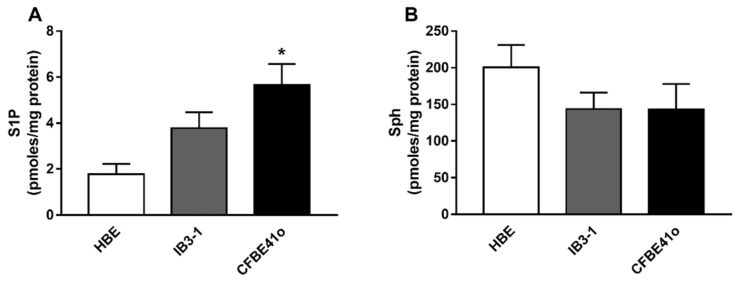
S1P and Sph expression in human bronchial epithelial immortalized cell lines. S1P (**A**) and Sph (**B**) content was evaluated by LC/MS analysis. Data are expressed as mean ± SEM. The descriptive statistics are extensively reported in the Supplementary Materials (Table S1). The results are reported as pmoles/mg protein. The statistical significance was evaluated by one-way ANOVA followed by Dunnett’s multiple comparisons test, when significant (*p* < 0.05). * *p* = 0.05 vs. HBE cell line. The cells were harvested at about 80% confluence and counted by Trypan Blue assay to assess that almost 85% of the cells were viable. All the cells used were from passages 5–10. HBE refers to healthy control cell lines while IB3-1 and CFBE41o to CF cell lines.

In order to evaluate the basal expression of S1P and Sph in CF vs. healthy human bronchial epithelial cell lines, we performed liquid chromatography separation coupled to mass spectrometry analysis of lipid extract from IB3-1 and CFBE41o vs. HBE control cells. As shown in Figure 1A, S1P intracellular concentration almost doubles in IB3-1 as compared to HBE, moving from 1.78 ± 0.44 to 3.78 ± 0.68 pmoles/mg protein while it reaches a threefold increase in CFBE41o (1.78 ± 0.44 vs. 5.66 ± 0.9 pmoles/mg protein; * *p* = 0.05). Conversely, the Sph level is reduced by 25% in both CF cell lines (Figure 1B).

### 3.2. Transcript Regulation of S1P Metabolism Enzymes in Human Bronchial Epithelial Cell Lines

**Figure 2:**
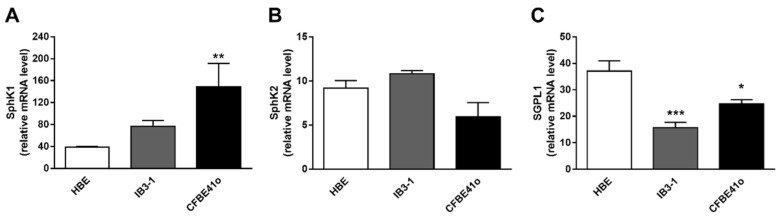
Transcript expression of the main synthesizing and degrading enzymes of S1P metabolism in human bronchial epithelial immortalized cell lines. RT-PCR analyzed the transcripts of the enzymes involved in S1P metabolism. Data are expressed as mean ± SEM. Descriptive statistics are extensively reported in the Supplementary Materials (Table S1). The results are reported as relative mRNA level of the target gene vs. GAPDH. The statistical significance was evaluated by one-way ANOVA followed by Dunnett’s multiple comparisons tests, when significant (*p* < 0.05). (**A**) SphK1 transcript modulation in control (HBE) vs. CF cell lines (IB3-1 and CFBE41o); ** *p* = 0.0036 vs. HBE. (**B**) SphK2 transcript modulation in control (HBE) vs. CF cell lines (IB3-1 and CFBE41o). (**C**) SGPL1 transcript modulation in control (HBE) vs. CF cell lines (IB3-1 and CFBE41o); * *p* = 0.0436; *** *p* = 0.0007 vs. HBE. The cells were harvested at about 80% confluence and counted by Trypan Blue assay to assess that almost 85% of the cells were viable. All the cells used were from passage 5–10.

The intracellular accumulation of S1P in CF human bronchial epithelial cell lines may derive from the alteration of the fine-tuned equilibrium between the enzymes that either synthesize or catabolize S1P. Thus, we used RT-PCR to analyze the transcript expression of the main enzymes involved in S1P metabolism. SphK1 and SphK2 phosphorylate sphingosine to generate intracellular S1P, the first enzyme being mainly cytosolic while the second also localizes to the ER, mitochondria, and nucleus. SphK1 mRNA expression (Figure 2A) is higher in both CF than control cells, (two-fold increase in IB3 vs. control HBE, *p* = ns; four-fold increase in CFBE vs. control HBE; ** *p* = 0.0036). Instead, SphK2 mRNA is similarly expressed in all the cell lines (Figure 2B). S1P is converted in its precursors by the enzyme SGPL1, while it entails a dynamic equilibrium with sphingosine, by the action of two phosphatases (SGPP1 and 2). We observed that SGPL1 mRNA significantly decreases in both CF vs. control cell lines, halving in IB3-1 cells (Figure 2C; *** *p* = 0.0007). On the contrary, SGPP1 and 2 mRNA show uneven modulation in CF compared to control cell lines. The two phosphatases’ transcripts rise in CFBE41o while diminishing in IB3-1 compared to HBE (Supplementary Materials Figure S1).

### 3.3. Expression and Intracellular Localization of the Spns2 Transporter in Human Bronchial Epithelial Cell Lines

**Figure 3:**
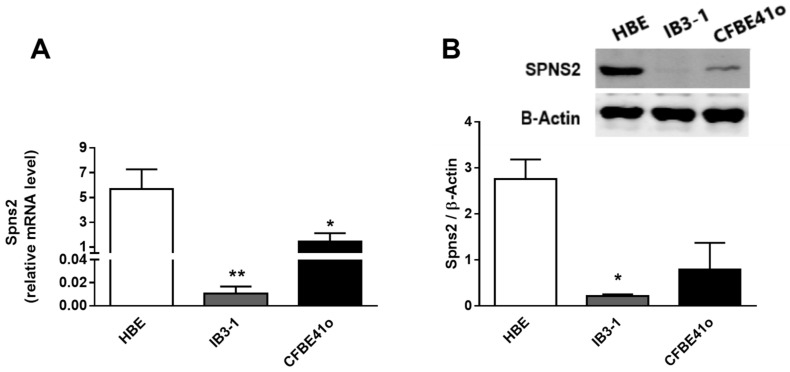
Transcript and protein expression of Spns2 transporter in human bronchial epithelial immortalized cell lines. (**A**) The mRNA expression of Spns2 transporter in control (HBE) vs. CF cell lines (IB3-1 and CFBE41o) as analyzed by RT-PCR. Data are expressed as mean ± SEM. Descriptive statistics are extensively reported in Supplementary Materials (Table S1). The results are reported as relative mRNA level of the target gene vs. GAPDH. The statistical significance was evaluated by one-way ANOVA followed by Dunnett’s multiple comparisons tests when significant (*p* < 0.05); * *p* = 0.0492; ** *p* = 0.0061 vs. HBE. (**B**) The expression of Spns2 (upper image) in control (HBE) vs. CF cell lines (IB3-1 and CFBE41o) as analyzed by Western blotting (* *p* = 0.0265). Ten micrograms of proteins were separated onto a 10% acryl/bis-acrylamide gel and β actin was used as a loading control (lower image). Spns2 was diluted at 1:500 and β actin 1:5000 in T-TBS. Data are expressed as mean ± SEM and results are normalized on β actin values. The Western blotting image is the most representative of four independent analyses. The complete Western blotting image is reported in the Supplementary Materials (Figure S3).

Spns2 is a transport protein that extrudes S1P out of the cells thus maintaining its intracellular homeostasis. As shown in Figure 3A, Spns2 mRNA is significantly downregulated in IB3-1 vs. HBE control cells (** *p* = 0.0061, more than 550-fold decrease), and the same trend is observed in CFBE41o, although to a lesser extent (* *p* = 0.05, four-fold decrease). Spns2 protein expression mirrors the mRNA modulation, decreasing in CF vs. HBE control cells. In addition, the transporter content significantly drops in IB3-1 cells, as shown by Western blotting (Figure 3B) and immunohistochemistry (Supplementary Materials Figure S2) analysis.

### 3.4. Expression of S1P and Sph in Human Bronchial Epithelial Primary Cells

**Figure 4:**
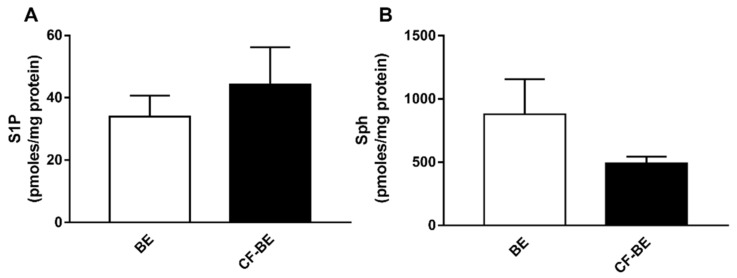
S1P and Sph expression in human bronchial epithelial primary cell lines. S1P (**A**) and Sph (**B**) content was evaluated by LC/MS analysis in human bronchial epithelial primary cells from CF patients (CF-BE, *n* = 4) and healthy subjects (BE, *n* = 4). The cells were harvested at about 70% confluence and counted by Trypan Blue assay to assess that almost 85% of the cells were viable. All the cells used were from passages 2–5. Data are expressed as mean ± SEM. Descriptive statistics are extensively reported in Supplementary Materials (Table S1). The results are reported as pmoles/mg protein. The statistical significance was evaluated by unpaired Student’s *t*-test.

In order to corroborate the data obtained in immortalized CF and control cell lines, we evaluated the basal expression of S1P and Sph in human bronchial epithelial primary cells from CF patients (CF-BE, *n* = 4) and healthy subjects (BE, *n* = 4). Accordingly, S1P (Figure 4A) accumulates in CF-BE as compared to BE cell lines (44.3 ± 11.8 vs. 33.9 ± 6.7 pmoles/mg protein) while Sph (Figure 4B) diminishes (492.1 ± 52.2 vs. 879.7 ± 275.5 pmoles/mg protein).

### 3.5. Transcript Regulation of S1P Metabolism Enzymes and Spns2 Transporter in Human Bronchial Epithelial Primary Cells

**Figure 5:**
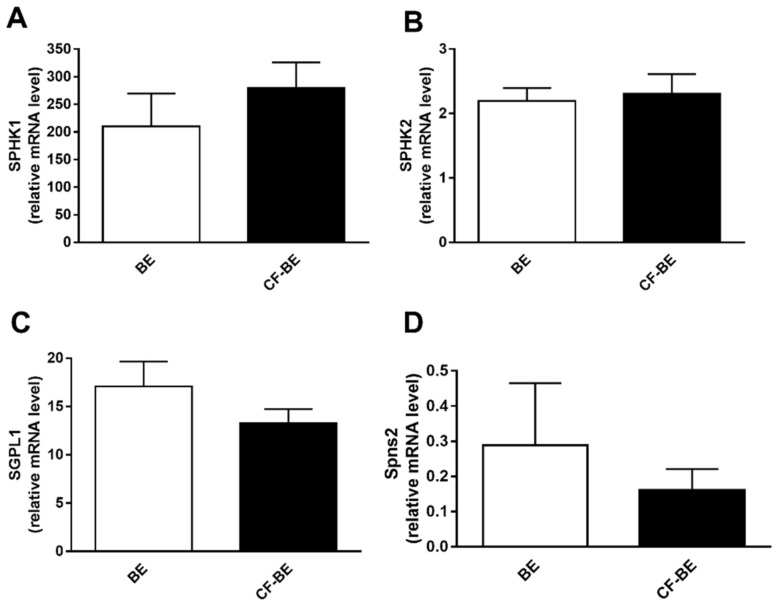
Transcript regulation of S1P metabolism enzymes and Spns2 transporter in human bronchial epithelial primary cells. The transcripts of the enzymes involved in S1P metabolism and the Spns2 transporter, were analyzed by RT-PCR in human bronchial epithelial primary cells from CF patients (CF-BE, *n* = 4) and healthy subjects (BE, *n* = 4). The cells were harvested at about 70% confluence and counted by Trypan Blue assay to assess that almost 85% of the cells were viable. All the cells used were from passage 2–5. Data are expressed as mean ± SEM. Descriptive statistics are extensively reported in Supplementary Materials (Table S1). The results are reported as relative mRNA level of the target gene vs. GAPDH. The statistical significance was evaluated by unpaired Student’s *t*-test. (**A**) SphK1 transcript modulation in control (BE) vs. CF primary cell lines (CF-BE). (**B**) SphK2 transcript modulation in control (BE) vs. CF primary cell lines (CF-BE). (**C**) SGPL1 transcript modulation in control (BE) vs. CF primary cell lines (CF-BE). (**D**) Spns2 transcript modulation in control (BE) vs. CF primary cell lines (CF-BE).

We further investigated the mRNA regulation of the S1P metabolism enzymes in human bronchial epithelial primary cells from CF patients (CF-BE, *n* = 4) and healthy subject (BE, *n* = 4). The mRNA of the enzymes involved in both the synthesis and the degradation of S1P shows the same trend observed in the immortalized cell line model, though not reaching statistical significance. SphK1 and SGPL1 mRNA (Figure 5A,C) are up- and down-regulated, respectively, in CF-BE compared to BE while SphK2 (Figure 5B) is unaffected. The transcripts’ expression of the two phosphatases, SGPP1 and 2, does not vary in CF-BE vs. BE cells (Supplementary Materials Figure S4). The Spns2 mRNA (Figure 5D) is about two-fold less abundant in CF-BE than in BE (0.16 ± 0.06 vs. 0.29 ± 0.18 expressed as relative mRNA level).

### 3.6. Expression of Spns2 Transporter in Human Lung

**Figure 6:**
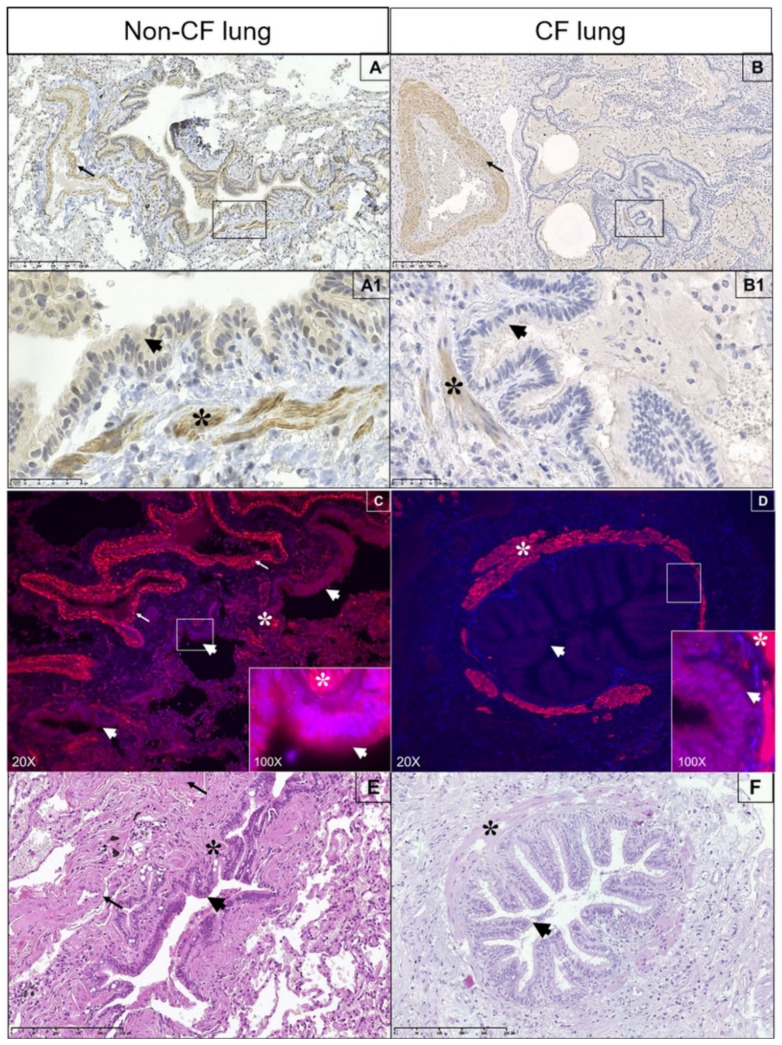
Expression of Spns2 transporter in human lung. The expression of Spns2 transporter in CF (*n* = 3) vs. non-CF (*n* = 3) human lung, was analyzed by immunohistochemistry (**A**,**A1**,**B**,**B1**) and immunofluorescence (**C**,**D**). (**A1**) represents the magnification of the inset in (**A**); (**B1**) represents the magnification of the inset in (**B**). Morphology of lung parenchyma positive in immunofluorescence is appreciable in consecutive sections stained with hematoxylin and eosin (**E**,**F**). Pulmonary parenchyma used as a control was sampled from surgical specimens for cancer distant from the lesion. Spns2 transporter stains positive in tunica media of vascular structure (thin arrows) and in muscularis mucosae of distal bronchioles (asterisks) and, to a lesser extent, in the cytoplasm of ciliated respiratory cells (thick arrows). Images are the most representative of different analyses.

We then analyzed the expression of Spns2 transporter in human lung from CF and non-CF patients, used as controls, by immunohistochemistry and immunofluorescence. In the non-CF lung, Spns2 is expressed in tunica media (thin arrows) of vascular structures, in muscularis mucosae (asterisks) of distal bronchiole (Figure 6A), and, to a lesser extent, in the cytoplasm of ciliated respiratory cells (thick arrows) (Figure 6A1, magnification of the inset in 6A). Pulmonary parenchyma of CF lung (Figure 6B) shows an overall attenuation of Spns2 expression versus non-CF tissue. In Figure 6B1, (magnification of the inset in 6B), Spns2 is decreased in ciliated respiratory cells (thick arrows) as compared to smooth muscle cells of the vascular network (thin arrows; asterisks) and distal airways. Accordingly, in the non-CF lung (Figure 6C), Spns2 immunofluorescence is appreciable in muscle cells and ciliated epithelium (inset). In CF lung (Figure 6D), Spns2 immunofluorescence, easily visible in bronchial muscle cells, is almost negative at the cytoplasmic level in the ciliated epithelium.

## 4. Discussion

**Figure 7:**
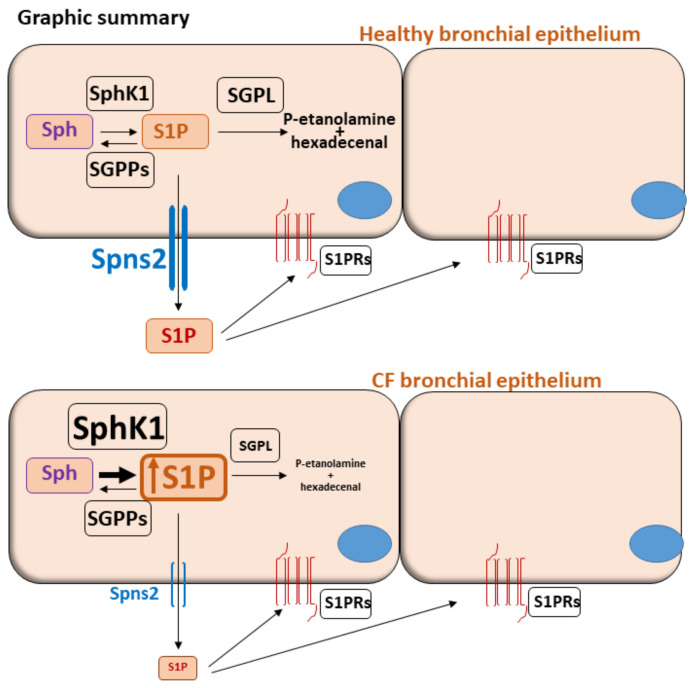
Graphic summary. Schematic representation of healthy and CF human bronchial epithelium. SphK1 and SGPPs finely tune the equilibrium between Sph and S1P while SGPL irreversible degrades S1P in its precursors, namely phospho(P)-etanolamine and hexadecenal. Once produced, S1P is extruded from the cell by Spns2 transporter and it activates autocrine and paracrine signaling by binding to G-protein coupled S1P receptors (S1PRs) on cell surfaces. In the CF model, S1P accumulation relies on SphK1 transcriptional up-regulation and SGPL down-regulation together with Spns2 impairment.

Dysregulation of SPL metabolism is known to characterize CF pathology. Our study investigates whether an altered S1P pathway could be part of the dysregulated SPL metabolism in CF and the actors involved in this scenario. It is widely described that SPL highly contribute to regulating pulmonary infections and inflammation in CF [3,4]. However, the main focus has been set on ceramide accumulation in the lungs of CF patients and on the ensuing unresolved inflammation [35,36,37]. Considering that S1P is a bioactive SPL mediator in cross-talk with the ceramide signaling pathway, we investigated the S1P metabolism in immortalized and primary human bronchial epithelial cells. Through mass spectrometry analysis, we found S1P accumulation in CF vs. healthy control cells. Previous evidences from the literature report conflicting data on S1P content in CF, mainly depending on the tissue or biologic samples analyzed. By our resultsan in vitro CF cell model showed that defective CFTR increases the synthesis of S1P, though the difference did not reach significance while Sph up-regulation was relevant [38]. As opposite, Veltman M. et al. observed a reduction of S1P content in total lung extract of unchallenged Cftrtm1EUR F508del CFTR mutant mice, thus leading to excessive inflammation responses in these mice [9]. In CF BALF (Broncho alveolar lavage fluid) from CFTR knockout mice, Xu Y. and colleagues described that S1P decreased as compared to wild type mice. In turn, this impaired pulmonary dendritic cells maturation and trafficking, thus affecting the clearance of pathogens from the CF lung [11]. In lung-transplanted CF patients, plasma levels of unbound S1P were lower than control subjects, and relevant differences were measured depending on the genotype of CF patients [10]. S1P concentration is normally elevated in blood and lymph, and low in tissues [39,40]. This gradient is finely tuned by a complex equilibrium between synthesizing and catabolizing enzymes, together with transporter proteins that regulate S1P passage across the plasma membrane. Here we describe that the S1P accumulation in CF cells relies on transcriptional up-regulation of SphK1 and simultaneous down-regulation of SGPL1, while SphK2 is unchanged. These results were replicated in both the immortalized and primary human bronchial epithelial cell lines, though the latter did not reach statistical significance probably for the high inter-individual variability among patients and the relatively small sample number. Compared to the control cell line, the different modulation of the transcripts of SGPP1 and 2 phosphatases in the two CF cell lines deserves further investigation. It has been reported that SphK1 expression is higher in the lung and heart, while SphK2 is present to a greater extent in the liver and spleen [41,42]. SphK1 is activated upon stimulation with cytokines [23], an inflammatory condition that nearly resembles the one observed in CF. Besides the autocrine or paracrine functions, S1P derived by SphK1 induces the NF-κB pathway activation in a receptor-independent manner, thus probably contributing to CF inflammation [14]. The high S1P accrual observed in our CF models cannot be dissipated since the Spns2 transporter, which normally extrudes S1P out of the cells, is reduced at the transcriptional and protein level. The regulation of Spns2 and its interplay with S1P metabolism machinery have already been described in several physiological and pathological conditions. Spns2 knockout mice display reduced lymphocytes number together with hearing loss and cataracts [22,43]. Mice knockout for Spns2 or with an endothelial-specific deletion show decreased T- and B-cells in the blood [21,44]. Loss of Spns2 influences the inflammatory responses and alters the humoral immune responses [17]. In a pathological setting of myocardial infarction, the bioactive peptide apelin enhances the secretion of S1P in lymphatic endothelial cells, by modulating the expression of Spns2 and SphK2 [45]. It has been demonstrated that the human lung highly expresses Spns2 transcripts compared to other investigated organs [46]. For the first time, we described a decrease in the expression of Spns2 in pulmonary parenchyma of patients affected by CF compared to control lung, mainly in the ciliated respiratory cells. Tran HB and colleagues [47] reported that Spns2 is reduced in bronchial epithelium of a mouse model of COPD (chronic obstructive pulmonary disease) compared to control animals with a strong positive correlation between Spns2 reduction and impairment of the phagocytic activity of macrophages. Moreover, they observed a significant Spns2 down-regulation in immortalized bronchial epithelial and primary nasal epithelial cells in response to cigarette-smoke exposition. S1P accumulation was described in the lung of Spns2 deficient mice even though its level was reduced in the plasma [18]. In lung cancer cells, Bradley E. and coworkers found that Spns2 expression influences the transcription of several enzymes involved in S1P metabolism, such as sphingosine kinases, S1P phosphatases and lyase 1. Per our results on CF human bronchial epithelial cells, S1P accumulates in Spns2 knockdown lung cancer cells because of enhanced S1P synthesis and reduced degradation [48]. In the lungs of βENaC mice, epithelial Spns2 and SphK2 downregulation at mucus obstruction sites lead to dysregulated S1P signaling [49]. In conclusion, we observed that S1P accrual in CF vs. healthy control cells is supported by SphK1 transcription activation and SGPL1 transcription reduction, along with deficient Spns2-driven extracellular transport of S1P, as below summarized in the graphic summary (Figure 7). As already reported in lung cancer and COPD, Spns2 expression might regulate S1P metabolism by influencing the finely tuned equilibrium between synthesizing and degrading enzymes in CF. Given the role of Spns2 in the killing activity of macrophages [47], we speculate that the disruption of Spns2-driven transport of S1P could also contribute to the known defective killing ability also in CF macrophages. It is worthy of investigating the relevance of this issue in the CF hyper-inflammation and we propose that S1P could play a role in the exacerbation of the pathology. Further studies on CF lungs are required to establish the relationship between the key enzymes and the transporters that establish the intra- and extracellular S1P content, thus providing a rationale for targeting the S1P pathway in CF.

## Supplementary Materials

The following are available online at https://www.mdpi.com/article/10.3390/biomedicines9091121/s1, Figure S1: Transcript expression of SGPP1 and SGPP2 enzymes in human bronchial epithelial immortalized cell lines, Figure S2: Spns2 transporter expression in control (HBE) vs. CF cell lines (IB3-1 and CFBE41o) analyzed by immunohistochemistry, Figure S3: Complete Western blotting image of Spns2 transporter, Figure S4: Transcript expression of SGPP1 and SGPP2 enzymes in human bronchial epithelial primary cells, Table S1: Descriptive statistics of the results.
