# Supplementary material for: Spns2 Transporter Contributes to the Accumulation of S1P in Cystic Fibrosis Human Bronchial Epithelial Cells
Source: Biomedicines. 2021 Aug 31;9(9):1121. doi: 10.3390/biomedicines9091121 (PMC8467635; doi:10.3390/biomedicines9091121)
Supplement: Supplementary file 1 [file biomedicines-09-01121-s001.zip › Supplementary Figures Table/Supplementary Figures' legends.pdf]

Figure S1. Transcript expression of SGPP1 and SGPP2 enzymes in human bronchial epithelial immortalized cell lines

The transcripts of the two phosphatases enzymes (SGPP1 and SGPP2) involved in S1P metabolism were analyzed by RT-PCR. Data are expressed as mean ± SEM. The results are reported as relative mRNA level of the target gene vs GAPDH. Descriptive statistics are extensively reported in Supplementary material (Table S1). The statistical significance was evaluated by one-way ANOVA followed by the Dunnett's multiple comparisons test when significant ($P < 0.05$). Panel **A**: SGPP1 transcript modulation in control (HBE) vs CF cell lines (IB3-1 and CFBE41o); *, $p = 0.0373$ vs HBE. Panel **B**: SGPP2 transcript modulation in control (HBE) vs CF cell lines (IB3-1 and CFBE41o).

Figure S2. Spns2 transporter expression in control (HBE) vs CF cell lines (IB3-1 and CFBE41o) analyzed by immunohistochemistry.

Brown immunoreactivity for Spns2 is appreciable in the cytoplasm of the different cell lines. A decreasing gradient of immunoreactivity is easily visible in HBE, CFBE41o and IB3-1 cell lines, by the corresponding mRNA and protein levels. The cells were harvested at about 80% confluence and counted by Trypan Blue assay to assess that almost 85% of the cells were viable. All the cells used were from passage 5-10.

Figure S3. Complete Western Blotting image. A single nitrocellulose membrane was cut in two fragments and either blotted with anti-Spns2 antibody (1:500 in T-TBS; upper fragment) or with anti-β-actin as loading control (1:500, in T-TBS; lower fragment). Spns2 protein has a molecular weight of 55 kDa; β-actin, 43 kDa. Muscle protein extracts (first lane) were used as positive control.

Figure S4. Transcript expression of SGPP1 and SGPP2 enzymes in human bronchial epithelial epithelial primary cells

The transcripts of the two phosphatases enzymes (SGPP1 and SGPP2) involved in S1P metabolism were analyzed by RT-PCR in human bronchial epithelial primary cells from CF patients (CF-BE, n=4) and healthy subjects (BE, n=4). Data are expressed as mean ± SEM. Descriptive statistics are extensively reported in Supplementary material (Table S1). The results are reported as relative mRNA level of the target gene vs GAPDH. The statistical significance was evaluated by unpaired Studen's t-test. Panel **A**: SGPP1 transcript modulation in control (BE) vs CF primary cell lines (CF-BE). Panel **B**: SGPP2 transcript modulation in control (BE) vs CF primary cell lines (CF-BE).

Table S1. Descriptive statistics of the results . The tables report the mean, SD, median, Q1, Q3, IQR, n of the results obtained in human bronchial epithelial immortalized and primary cell lines.
