# Supplementary material for: Spns2 Transporter Contributes to the Accumulation of S1P in Cystic Fibrosis Human Bronchial Epithelial Cells
Source: Biomedicines. 2021 Aug 31;9(9):1121. doi: 10.3390/biomedicines9091121 (PMC8467635; doi:10.3390/biomedicines9091121)
Supplement: Supplementary file 1 [file biomedicines-09-01121-s001.zip › Supplementary Figures Table/Table S1.pdf]

	S1P Figure 1A		
	HBE	IB3-1	CFBE41o
Mean	1,78365	3,78455433	5,66072867
SD	1,47420496	1,68692197	2,22133909
Median	1,7835	3,826	5,6
Q1	1,012025	2,73946525	5,0914355
Q3	2,555125	4,21452925	6,3983435
IQR	1,5431	1,475064	1,306908
n	4	6	6

	Sph Figure 1B		
	HBE	IB3-1	CFBE41o
Mean	200,442717	152,898887	143,275822
SD	74,8430775	58,6071464	84,5968856
Median	196,52295	146,0496	103,79378
Q1	135,655625	100,003095	86,21592
Q3	261,835	205,5225	177,6385
IQR	126,179375	105,519405	91,42258
n	6	6	6

	SphK1 Figure 2A		
	HBE	IB3-1	CFBE41o
Mean	38,652728	77,089188	149,0778
SD	3,87313547	23,8112406	73,6917612
Median	37,92709	73,2964	110,8648
Q1	36,5898	64,907	106,60305
Q3	42,00993	82,19993	172,44605
IQR	5,42013	17,29293	65,843
n	5	5	3

	SphK2 Figure 2B		
	HBE	IB3-1	CFBE41o
Mean	9,24516	10,81858	5,9365225
SD	1,45022266	1,57319947	2,67483857
Median	9,3498	10,7528	5,0695
Q1	9,2396	10,2459	4,09244
Q3	9,3988	11,4845	6,9135825
IQR	0,1592	1,2386	2,8211425
n	5	5	4

	SGPL1 Figure 2C		
	HBE	IB3-1	CFBE41o
Mean	37,10802	15,66126	24,6485667
SD	8,79111409	4,71109136	2,86236204
Median	39,1172	15,905	24,4492
Q1	32,1379	12,5618	23,17015
Q3	39,7976	17,1237	26,0273
IQR	7,6597	4,5619	2,85715
n	5	5	3

	Spns2 Figure 3A		
	HBE	IB3-1	CFBE41o
Mean	5,6699566	0,0108375	1,4368928
SD	3,59757426	0,01347129	1,17994936
Median	5,326801	0,00791054	1,3499
Q1	3,2359	0,0026	0,8263737
Q3	7,406262	0,00794392	2,0039155
IQR	4,170362	0,00534392	1,1775418
n	5	5	3

	Spns2 Figure 3B		
	HBE	IB3-1	CFBE41o
Mean	2,5611875	0,1920714	0,7968095
SD	1,07643985	0,16487704	1,09488904
Median	2,3964205	0,2502142	0,2104285
Q1	1,87977575	0,1281071	0,16521425
Q3	3,07783225	0,2851071	1,13521425
IQR	1,1980565	0,157	0,97
n	4	3	3

	SGPP1 Figure S1		
	HBE	IB3-1	CFBE41o
Mean	24,4697425	19,45721	64,9266567
SD	7,16536317	5,45198168	31,3776577
Median	23,67921	18,3201	75,86288
Q1	21,54443	17,22154	52,703635
Q3	26,6045225	20,56104	82,61779
IQR	5,0600925	3,3395	29,914155
n	4	5	3

	SGPP2 Figure S1		
	HBE	IB3-1	CFBE41o
Mean	3,221024	0,747748	33,5662767
SD	1,87173617	1,33377981	24,4343283
Median	3,82442	0,15954	32,15035
Q1	1,46897	0,14825	21,010525
Q3	4,35684	0,16686	45,414065
IQR	2,88787	0,01861	24,40354
n	5	5	3

	S1P Figure 4A		Sph Figure 4B		SPHK1 Figure 5A		SPHK2 Figure 5B	
	BE	CF-BE	BE	CF-BE	BE	CF-BE	BE	CF-BE
Mean	33,88379	44,27338	879,685	492,1915	210,192	280,2045	2,19575	2,30575
SD	10,04781	17,31776	381,1091	87,86609	119,095	92,26924	0,399814	0,61495
Median	35,39569	44,27554	872,0551	499,278	170,5665	286,675	2,2655	2,481
Q1	28,58774	30,97387	738,1675	429,2645	142,0693	232,0355	2,062	2,066
Q3	40,69174	57,57504	1013,573	562,205	238,6893	334,844	2,39925	2,72075
IQR	12,104	26,60117	275,4051	132,9405	96,62	102,8085	0,33725	0,65475
n	4	4	4	4	4	4	4	4

	SGPL1 Figure 5C		SPNS2 Figure 5D		SGPP1 Figure S4		SGPP2 Figure S4	
	BE	CF-BE	BE	CF-BE	BE	CF-BE	BE	CF-BE
Mean	17,05025	13,279	0,289	0,1615	28,85445	24,30733	5,340371	4,146111
SD	5,201455	2,931284	0,351646	0,118632	8,960104	8,495972	7,140016	1,693533
Median	16,2445	12,644	0,155	0,1245	27,51598	21,25972	2,28463	4,504247
Q1	12,93475	12,032	0,0695	0,1065	22,14028	19,03395	1,270432	3,292963
Q3	20,36	13,891	0,3745	0,1795	34,23015	26,5331	6,354569	5,357395
IQR	7,42525	1,859	0,305	0,073	12,08987	7,499154	5,084138	2,064432
n	4	4	4	4	4	4	4	4
