# Supplementary figures and images for: Spns2 Transporter Contributes to the Accumulation of S1P in Cystic Fibrosis Human Bronchial Epithelial Cells
Source: Biomedicines. 2021 Aug 31;9(9):1121. doi: 10.3390/biomedicines9091121 (PMC8467635; doi:10.3390/biomedicines9091121)

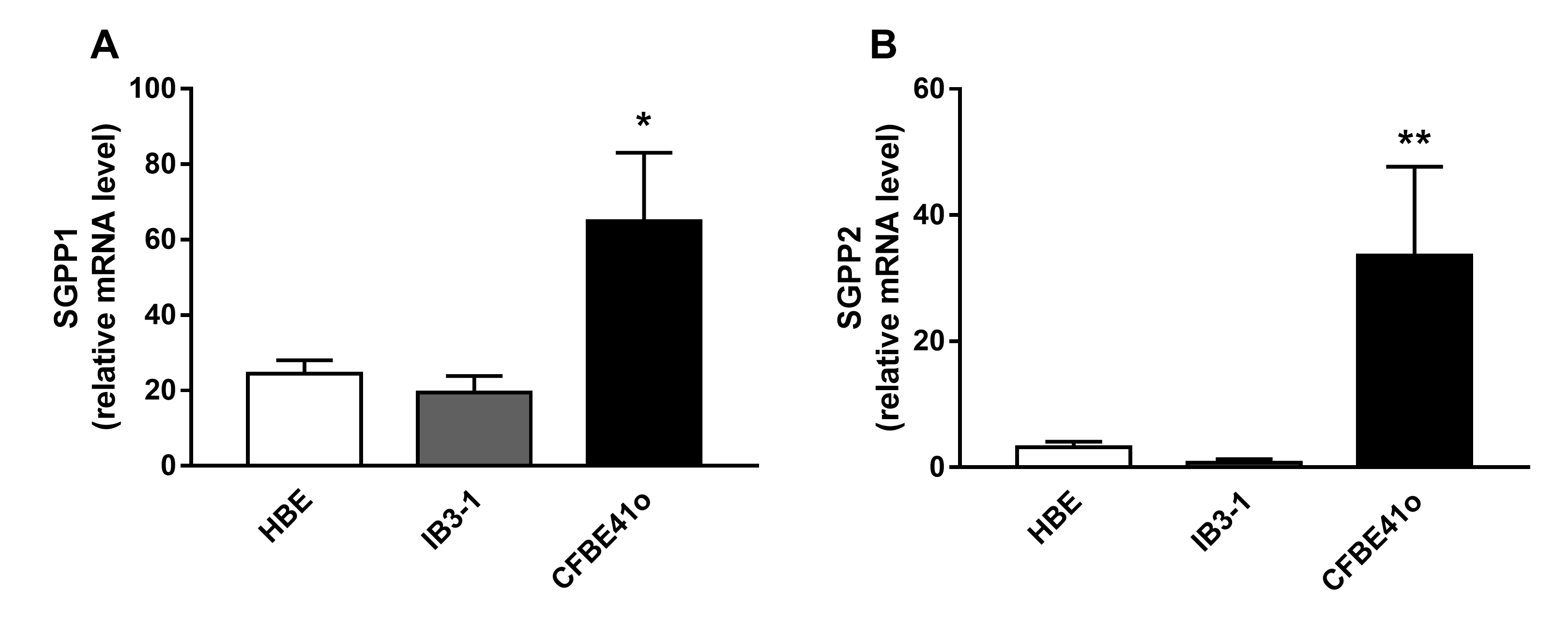

Supplement: Supplementary file 1 [file biomedicines-09-01121-s001.zip › Supplementary Figures Table/FigureS1.tif]

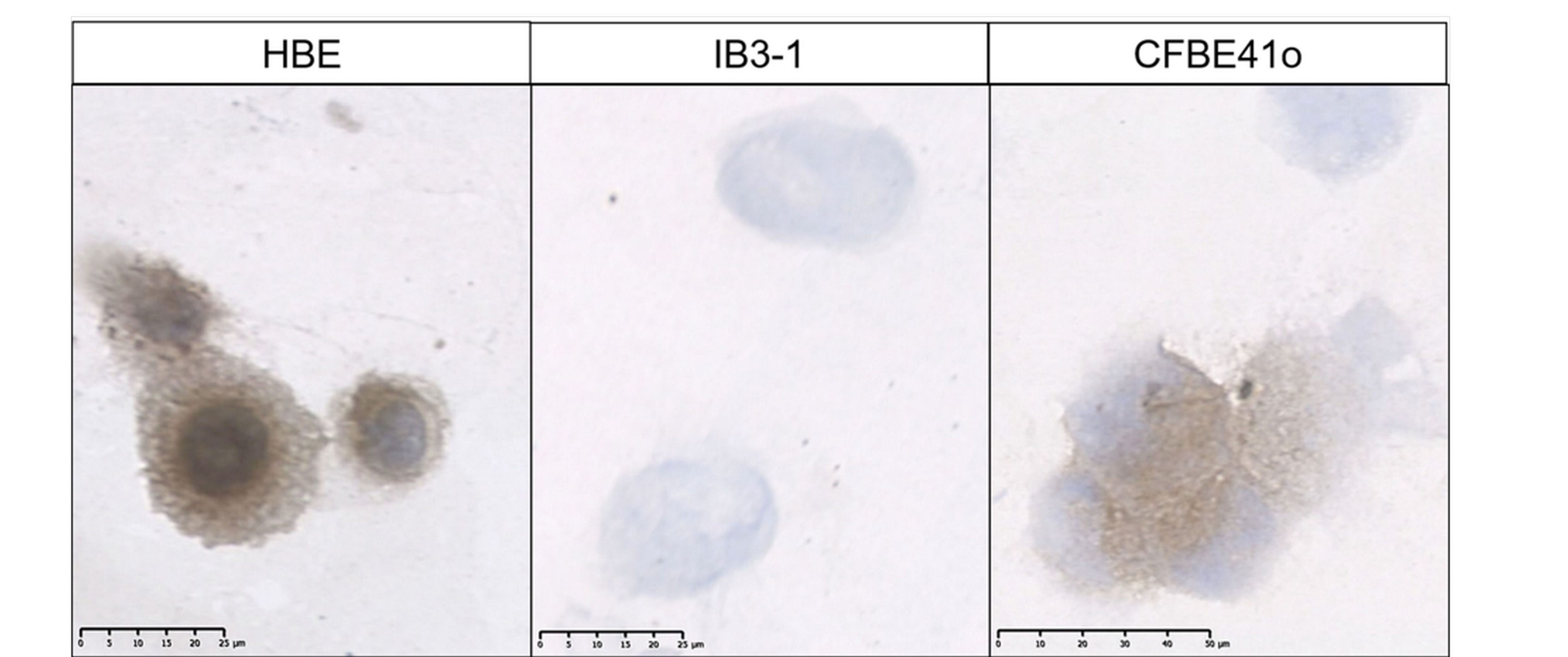

Supplement: Supplementary file 1 [file biomedicines-09-01121-s001.zip › Supplementary Figures Table/FigureS2.tif]

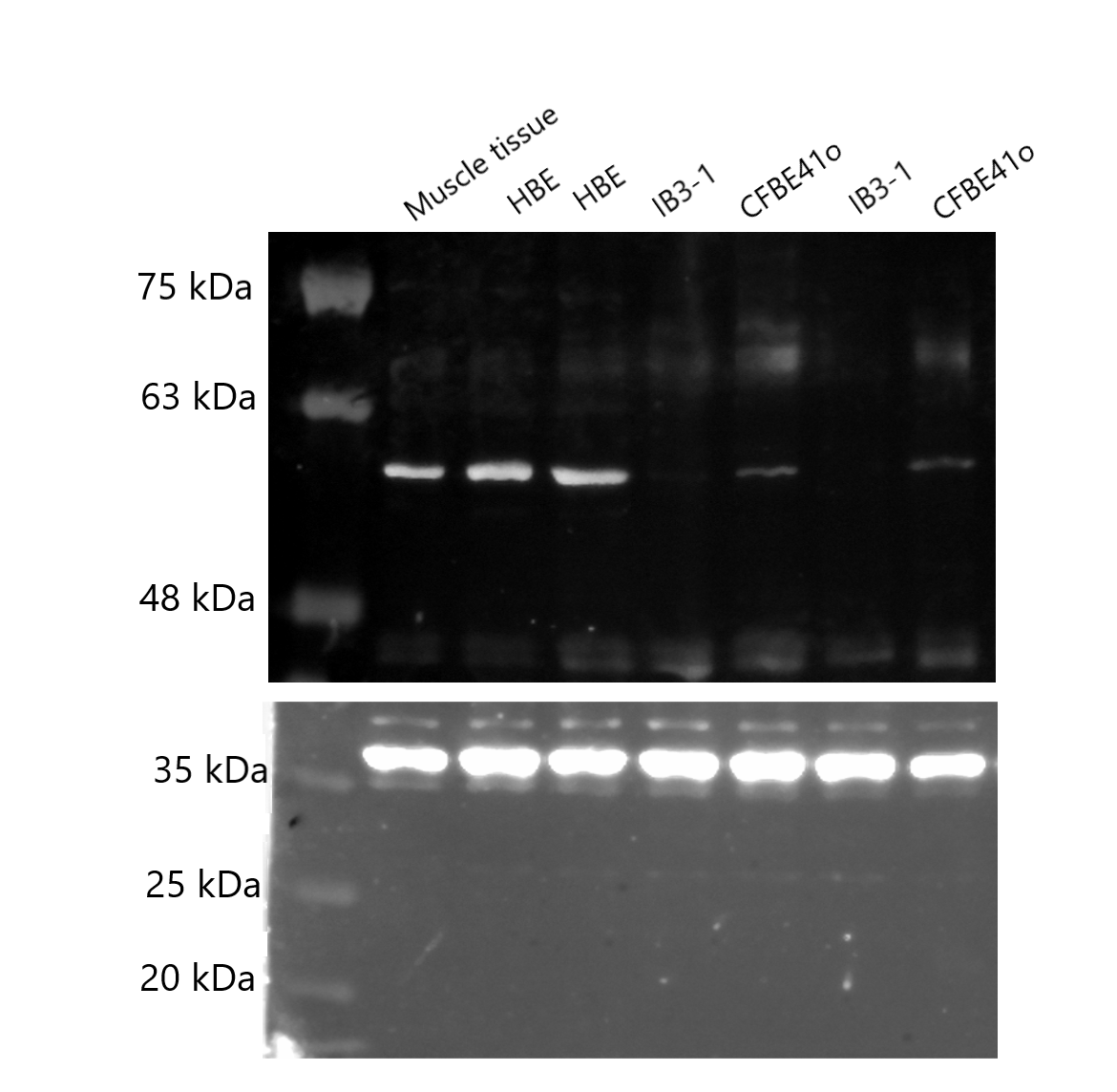

Supplement: Supplementary file 1 [file biomedicines-09-01121-s001.zip › Supplementary Figures Table/FigureS3.tiff]

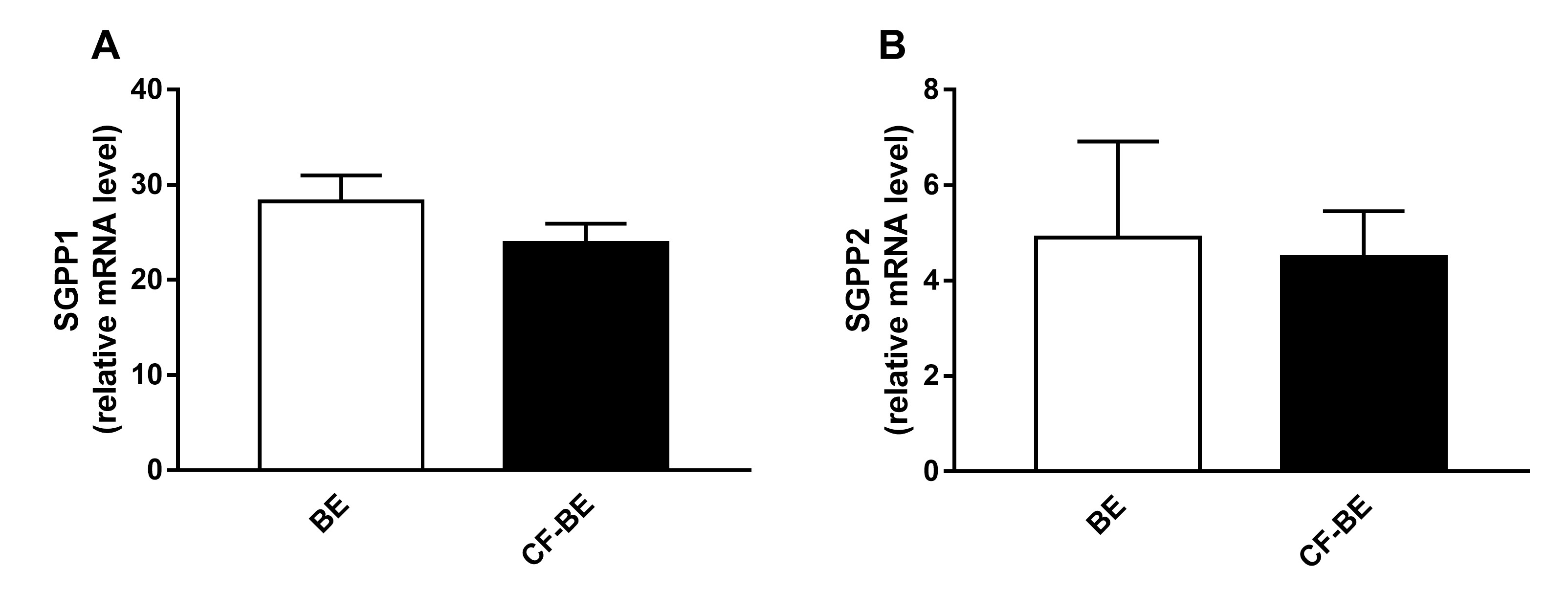

Supplement: Supplementary file 1 [file biomedicines-09-01121-s001.zip › Supplementary Figures Table/FigureS4.tif]
